# Microsatellite instability in metaplasia-dysplasia-adenocarcinoma sequence of Barrett esophagus: a retrospective study

**DOI:** 10.3325/cmj.2018.59.100

**Published:** 2018-06

**Authors:** Pave Markoš, Iva Brčić, Luka Brčić, Jasminka Jakić-Razumović, Roland Pulanić

**Affiliations:** 1 Division of Gastroenterology and Hepatology, University Hospital Center Zagreb, Zagreb, Croatia; 2 Clinical Department for Pathology and Cytology, University Hospital Center Zagreb, Zagreb School of Medicine, Zagreb, Croatia

## Abstract

**Aim:**

To analyze the loss of mismatch repair (MMR) system protein expression in metaplasia-dysplasia-adenocarcinoma sequence of Barrett esophagus (BE).

**Methods:**

This study retrospectively analyzed the data from 70 patients with pathohistological diagnosis of BE or esophageal adenocarcinoma (EAC) treated at the Clinical Department of Pathology and Cytology, University Hospital Center Zagreb, from January 2009 to January 2011. Patients were divided into three groups: BE without dysplasia (22 patients), BE with dysplasia (37 patients), and EAC (11 patients). Immunohistochemical expression of MutL homologue 1 (MLH1), MutS homologue 2 (MSH2), postmeiotic segregation increased 2 (PMS2), and MutS homologue 6 (MSH6) of DNA MMR system was measured and compared with tumor protein p53 expression.

**Results:**

A total of 81.8% and 81.8% patients with EAC, 32.4% and 35.1% patients with dysplasia, and 50% and 54.5% patients without dysplasia had loss of MLH1 and PMS2 expression, respectively. Patients with EAC and patients with dysplasia did not have loss of MSH2 and MSH6 expression, and 18.2% patients without dysplasia had loss of MSH2 and MSH6 expression. There was a strong positive correlation between MLH1 and PMS2 expression (Spearman ρ 0.97; *P* < 0.001) and between MSH2 and MSH6 expression (Spearman ρ 0.90, *P* < 0.001) in the entire sample and in all BE groups. No significant correlations of MLH1 and PMS2 with p53 expression were found, except in dysplasia group (φ 0.402, *P* = 0.030 for MSH1; φ 0.371, *P* = 0.042 for PMS2).

**Conclusion:**

Although we demonstrated considerable loss of MLH1 and PMS2 expression in BE-associated carcinoma sequence, due to the retrospective study design and low number of patients we cannot conclude that MLH1 and PMS2 can be used as biomarkers for patient surveillance and therapy-making decisions.

Oxford Centre for Evidence-based Medicine level of evidence: 3

Barrett esophagus (BE) is considered a main premalignant condition and the most important risk factor for esophageal adenocarcinoma (EAC). In this condition, stratified squamous non-keratinized epithelium is replaced with metaplastic columnar epithelium and specialized goblet cells (1). BE prevalence in adult population is about 1.6% (2), and patients with BE have a 40-125 times higher risk of EAC than general population (1,3). In the last 30 years, EAC incidence among white men has risen by more than 350%, with overall poor prognosis – less than 50% of patients survive one year after the diagnosis (4). BE-associated EAC develops through a multi-step process, intestinal metaplasia-dysplasia-adenocarcinoma, involving different triggers of neoplastic progression, such as chromosomal abnormalities, genetic, and epigenetic events and environmental factors (5). However, only 0.7% patients with BE per year actually develop EAC (6). There are still no accepted clinical parameters or valid biomarkers (besides dysplasia) identifying the patients at higher EAC risk, who need frequent surveillance and early intervention. Intestinal metaplasia-dysplasia-adenocarcinoma sequence is similar to adenoma-carcinoma sequence in colorectal carcinogenesis and is characterized by sequential accumulation of genetic alterations (5).

One of the most important causes of colorectal carcinogenesis are DNA mismatch repair (MMR) system mutations. MMR is a sensory system that scans DNA, detects and removes nucleotide mispair, and activates DNA polymerase that repeats the synthesis. Its inactivity causes microsatellites shortening, microsatellite instability (MSI) phenotype, and immunohistochemical loss of MMR protein expression (7). MSI-high (MSI-H) is the presence of mutations at 2 or more of the 5 consensus microsatellite sequences and MSI-low (MSI-L) is the presence of mutations at only 1 microsatellite sequence.

The most important protein families in DNA MMR system are the MutS homologue (MSH) and MutL homologue (MLH). MSH is an obligatory partner of the MutS protein family, which dimerizes with two other family members: MSH6 (forming MutSα) or MSH3 (forming MutSβ) (8). MLH1 is an obligatory partner of the MutL protein family, which dimerizes with postmeiotic segregation increased 2 (PMS2), postmeiotic segregation increased 1 (PMS1), or MtuL homologue 3 (MLH3). Loss of MSH2 or MLH1 leads to a complete loss of DNA MMR activity and accelerated accumulation of DNA synthetic errors as the cells proliferate (9-11).

Most studies indicate that the MSI-H status is not the major tumorigenic pathway in BE-associated EAC, and the prevalence of MSI-H and MSI-L is approximately 5%-10% (12-14). Defective MMR has been established as a tumorigenic pathway in about 15%-20% of sporadic gastrointestinal tumors and endometrial cancers (15). The vast majority of MSI-H colorectal cancers is associated with sporadic methylation of the hMLH1 promoter (16). Germline mutations in MMR genes also predispose MSI-H hereditary nonpolyposis colorectal cancer (HNPCC) (17). Several studies have reported that defective MMR occurs in 4.7% of BE-associated adenocarcinomas (12,18,19).

However, little is known about defective MMR system in metaplasia-dysplasia-carcinoma sequence. Our hypotheses were that loss of protein expression would be higher in advanced stages of BE (dysplasia and EAC) than in non-dysplastic BE and that we can use MSI immunohistochemistry as a biomarker for disease progression. Thus, the primary aim of this study was to evaluate the presence of MMR system mutations by using immunohistochemical analysis of MSI protein expression in different BE stages and EAC. The second aim was to assess whether immunohistochemistry of MSI protein expression can be used as a potential biomarker for patient surveillance and compare it to the most commonly used marker, tumor protein p53.

## Materials and methods

### Patients

In this single-center retrospective study, data were collected from the Clinical Department of Pathology and Cytology, University Hospital Center Zagreb, between January 2009 and January 2011. We identified 70 patients (52 men) diagnosed with BE or EAC. Pathohistological diagnosis was made by analyzing either biopsy samples of esophageal mucosa taken during upper gastrointestinal endoscopy according to the Seattle protocol (2) or surgical specimens (for the majority of EAC patients). Patients with gastroesophageal reflux disease, squamocellular carcinoma, and endoscopic suspicion of BE without adequate pathohistological verification were not taken into consideration. We divided the patients into three groups: BE without dysplasia (22 patients), BE with dysplasia (37 patients), and EAC (11 patients). The sample of only 3 patients with high grade dysplasia (HGD) was too small to be included in statistical analysis, so the group with dysplasia consisted of both low grade dysplasia (LGD) and HGD patients. The study did not include repeated biopsies from the same patient. Ethical approval was obtained from the Ethics Committee of the University Hospital Center Zagreb (class 8.1-12/45-3, No. 02/21-LJH, Zagreb, May 28, 2012).

### Histologic evaluation and immunohistochemistry

Histopathological specimens were retrieved from the Department for Pathology and Cytology, University Hospital Center Zagreb. Formalin-fixed and paraffin-embedded endoscopic specimens were sliced into 4-μm-thick serial-step sections. Immunohistochemical stains were performed using the standard streptavidin-biotin-peroxidase procedure. Primary monoclonal antibodies against MLH1 (clone ES05, diluted 1:50, Novocastra, Newcastle, UK), MSH2 (clone 25D12, diluted 1:40, Novocastra), MSH6 (clone PU29, diluted 1:100, Novocastra), PMS2 (clone M0R4G, diluted 1:100, Novocastra), and p53 (clone DO-7, dilution 1:25, Dako, Glostrup, Denmark) were used. The sections were deparaffinized, rehydrated, and washed in xylene, graded alcohols, and distilled water. Endogenous peroxide activity was blocked by incubation with 3% H_2_O_2_. The sections were placed in 10 mM citrate buffer at pH 6, and microwave antigen retrieval was performed. The EnVision (Dako) system was a secondary detection tool, and diaminobenzidine tetrahydrochloride served as a chromogen. Staining was performed using an automatized immunostainer (Dako). The slides were counterstained with hematoxylin. Nuclear staining of non-neoplastic epithelial esophageal cells and lymphocytes was used as internal positive control.

Protein expression of MLH1, MSH2, MSH6, and PMS2 was defined as negative or positive. Negative protein expression (abnormal immunohistochemistry) was defined as a complete absence of nuclear staining within the tumor cells in the presence of positive labeling in internal non-neoplastic cells. Positive expression was defined as unequivocal nuclear staining in neoplastic cells. Nuclear p53 staining was defined as positive (>10% of the neoplastic cells) or negative (<10% of the neoplastic cells). The stains on biopsies and resections were reviewed independently and blindly by two pathologists (IB and LB).

### Statistical analysis

Normality of quantitative variables distribution was assessed by Shapiro Wilk test when the sample size was under 30 and Kolmogorov-Smirnov test when the sample size was over 30. The significance of the relationship between two variables with more than two categories (difference between protein expression in three stages of BE) was analyzed with χ^2^ test. Statistical significance between two binary variables (differences in protein expression between two proteins) was analyzed with Fisher exact test. Correlations between the variables were tested using Phi correlation analysis for binary outcomes and Spearman ρ correlation analysis. Statistical significance of between-group differences for non-normally distributed variables was assessed using Mann-Whitney U test. We used two-sided significance tests in all cases, with the level of statistical significance set at 0.05. All statistical analyses were performed using SPSS 17.0 package (SPSS Inc., Chicago, IL, USA owned by Biometrika Healthcare Research).

## Results

**Figure 1:**
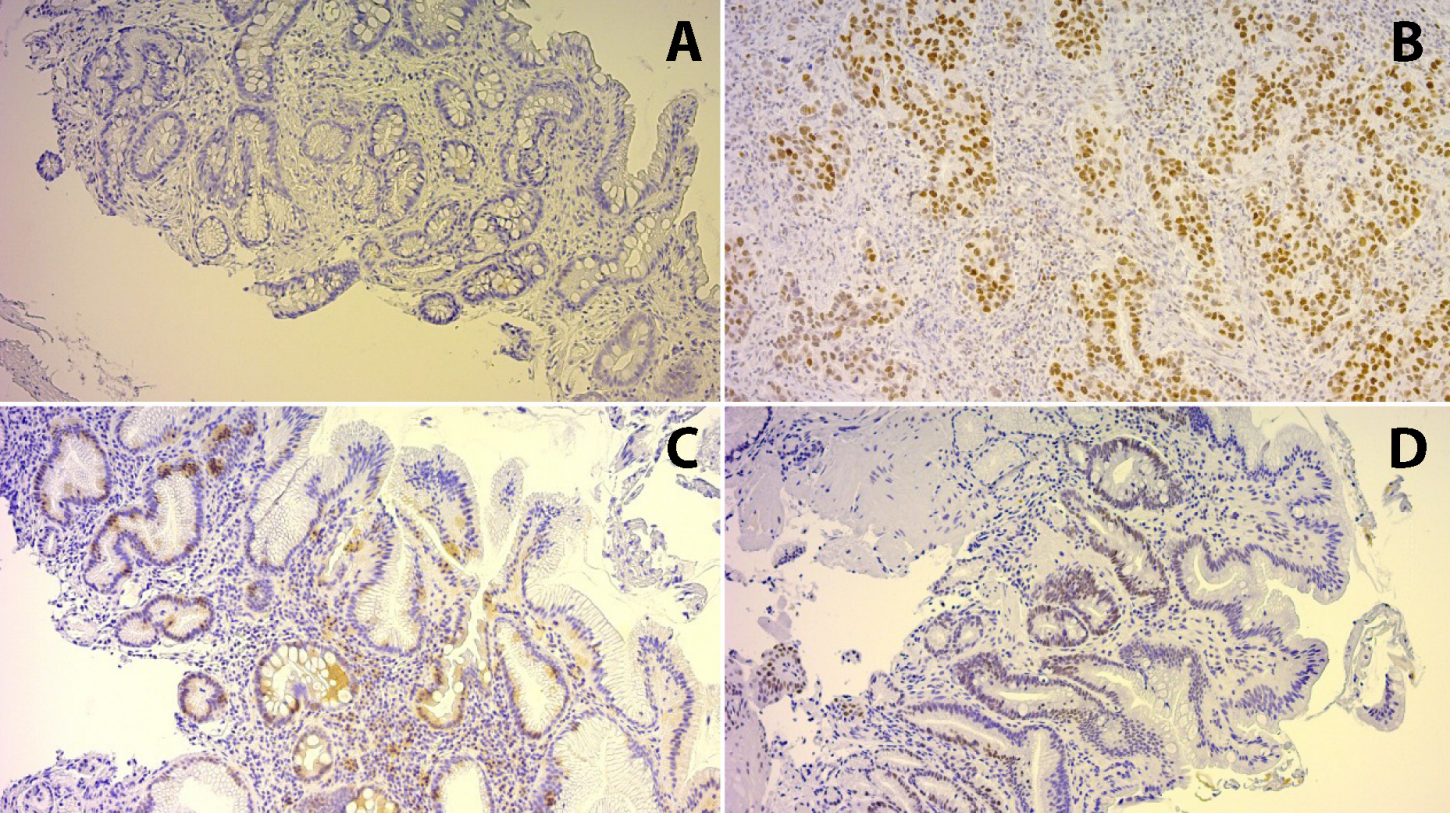
Immunohistochemistry of microsatellite instability in different stages of Barrett esophagus. (**A**) Loss of MutL homologue 1 expression in Barrett esophagus without dysplasia; (**B**) positive MutS homologue 2 expression in esophageal adenocarcinoma; (**C**) loss of MutL homologue 1 expression in Barrett esophagus with low grade dysplasia; (**D**) positive MutL homologue 1 expression in Barrett esophagus without dysplasia.

The mean age ± standard deviation (SD) of the patients was 63.2 ± 10.77 years. We found no significant differences between any protein expression and sex, age, or different BE groups (χ^2^ test). A total of 81.8% and 81.8% patients with EAC, 32.4% and 35.1% patients with dysplasia, and 50% and 54.5% patients without dysplasia had loss of MLH1 and PMS2 expression, respectively (Figure 1). Significant differences in MLH1, MSH2, PMS2, and MSH6 expression were found among the three patient groups (Table 1). Patients with EAC and patients with dysplasia did not have loss of MSH2 or MSH6 protein expression, while patients without dysplasia did (Table 1). There was a strong significant positive correlation between MLH1 and PMS2 expression (Spearman ρ 0.97, *P* < 0.001) in the whole sample and in all BE groups (Table 2). The same was observed for MSH2 and MSH6 expression (Spearman ρ 0.90, *P* < 0.001) (Table 3).

**Table 1:** Protein expression in three groups of Barrett esophagus patients

	No. of Barrett esophagus patients				
Protein expression	without dysplasia (n = 22)	with dysplasia (n = 37)	with esophageal adenocarcinoma (n = 11)	*P*	effect
**MutL homologue 1**					
lost	11	12	9	0.014	0.330*
positive	11	25	2		
**MutS homologue 2**					
lost	4	0	0	0.019	0.342*
positive	18	37	11		
**Postmeiotic segregation increased 2**					
lost	12	13	9	0.019	0.318*
positive	10	24	2		
**MutS homologue 6**					
lost	4	0	0	0.018	0.342*
positive	18	37	11		
**Tumor protein p53**					
negative	3	5	2	>0.999	
positive	19	32	9		

*χ^2^ test.

**Table 2:** Correlation between MutL homologue 1 and postmeiotic segregation increased 2 protein expression in patients with Barret esophagus

Patient groups	Spearman ρ	*P*
All (n = 70)	0.970	<0.001
Without dysplasia (n = 22)	0.952	<0.001
Dysplasia (n = 37)	0.964	<0.001
Esophageal adenocarcinoma (n = 11)	1.000	

**Table 3:** Correlation between MutS homologue 2 and MutS homologue 6 protein expression in patients with Barret esophagus

Patients	Spearman ρ	*P*
All (n = 70)	0.907	<0.001
Without dysplasia (n = 22)	0.955	<0.001
Dysplasia (n = 37)	0.892	<0.001
Esophageal adenocarcinoma (n = 11)	0.831	0.002

P53 expression was positive in 19 of 22 BE patients without dysplasia, 32 of 37 BE patients with dysplasia, and 9 of 11 patients with EAC. We found no significant correlations of MLH1 and PMS2 with p53 expression (Table 4), except in dysplasia group (φ 0.402, *P* = 0.030 for MSH1; φ 0.371, *P* = 0.042 for PMS2) (Table 5). We did not find a correlation of MSH2 and MSH6 with p53 expression in non-dysplasia group, and in dysplasia and EAC group no analysis was made owing to a lack of MSH2 and MSH6 expression.

**Table 4:** Correlations of MutL homologue 1, postmeiotic segregation increased 2, MutS homologue 2, and MutS homologue 6 protein expression with tumor protein p53 expression in patients with Barret esophagus

	No. of patients with tumor protein p53			
Protein expression	negative (n = 10)	positive (n = 60)	P*	φ correlation coefficient
**MutL homologue 1**				
lost	8	24	0.036	0.281
positive	2	36		
**Postmeiotic segregation increased 2**				
lost	8	26	0.043	0.257
positive	2	34		
**MutS homologue 2**				
lost	0	4	>0.999	-0.101
positive	10	56		
**MutS homologue 6**				
lost	0	4	>0.999	-0.101
positive	10	56		

*Fisher exact test for dichotomous variables.

**Table 5:** Correlations of MutL homologue 1 and postmeiotic segregation increased 2 protein expression with tumor protein p53 expression in the dysplasia group of patients with Barret esophagus

	No. of patients with tumor protein p53 expression (n = 37)			
Protein expression	negative (n = 5)	positive (n = 32)	P*	φ correlation coefficient
**MutL homologue 1**				
lost	4	8	0.030	0.402
positive	1	24		
**Postmeiotic segregation increased 2**				
lost	4 (	9	0.042	0.371
positive	1	23		

*Fisher exact test for dichotomous variables.

## Discussion

Our study demonstrated differences in the MLH1, MSH2, MSH6, and PMS2 expression in metaplasia-dysplasia-EAC sequence of BE. Our hypothesis that loss of protein expression would be higher in advanced stages of BE was refuted, since we found loss of MLH1/PMS2 expression in all three groups, most commonly in EAC group but also in 50% of patients without dysplasia. Although we clearly demonstrated the presence of the MMR system mutations in BE-associated carcinoma sequence, we cannot conclude that they can be used as biomarkers for therapy making decisions in non-dysplastic BE.

It is important to timely identify the patients who will progress to EAC and conditions with high malignant potential (like HGD and early EAC) (20). Dysplasia, a surrogate endpoint for EAC, is currently assessed in periodic endoscopic biopsies. However, a large number of biopsies has to be performed because of sampling errors (21), and biopsies are characterized by intra- and inter-observer discrepancies in lesion grading and staging (22).

LGD has a low progression rate to EAC, pathohistological diagnosis of LGD has low reproducibility, and LGD is usually not detected in control endoscopy (23-25). HGD has varying five-year cumulative incidence rates of progression (24,26). This is why many new biomarkers are being evaluated for better risk stratification of cancer development (27). Such biomarkers need to be part of causal pathway to EAC, have substantial predictive power to distinguish between the patients who will and will not develop EAC, and be easily and objectively measured. Although most biomarkers need to be further evaluated, most extensively documented ones are aneuploidy status, and *p16 and p53* gene abnormalities and allelic losses (27). Out study evaluated the role of MSI protein expression as a potential new, cheap, and widely available biomarker for assessing disease progression.

Basic demographic characteristics of our patients are similar to Western-European population (2-4). Most of the patients (3/4) were men, but differences in sex distribution between different patient groups were not significant, probably due to the small number of patients. Male sex is a well-known risk factor for EAC (28,29), but there are no sufficient data on patients’ age (30). In our study, patients without dysplasia were the youngest (mean age 58.5 years), however the difference between patients in dysplasia and EAC group was not significant. This could be explained by much faster progression to EAC in dysplastic than in non-dysplastic BE. We diagnosed 11 patients with EAC in two years (15% of our population), which is in accordance with a rising EAC incidence in the world (1,3,4).

Loss of MLH1/PMS2 expression in 81% of patients with EAC in our study is contrary to the low prevalence (3.5% to 6.6%) of MSI-H BE-associated EAC found in other studies (12,31). However, Cai and Liu (32) have shown MSI in almost 65% of their patients with EAC and in adjacent metaplastic and dysplastic tissue. We did not perform immunohistochemistry staining in the same patient with different pathohistological analyses, believing that the patients with MLH1/PMS2 loss of expression and metaplasia or dysplasia probably need closer follow up than patients with normal protein expression.

HNPCC is characterized by defective MMR (80%-90% germline mutations of MLH1 and MSH2), but the majority of sporadic MSI tumors are caused only by gene silencing through hypermethylation of MLH1 promoter, and somatic inactivation of MSH2 seems to be very rare (33,34). Usually loss of MSH2 expression strongly suggests a tumor associated with HNPCC. This could explain no significant loss of MSH2/MSH6 expression in our sample. Patients with EAC and those with dysplasia did not have loss of MSH2/MSH6 expression, so we can conclude that EAC is a sporadic tumor, rather than a tumor associated with HNPCC, and that only MLH1/PMS2 mutation is of interest in patient surveillance.

There are no data on the relationship between age and sex and biomarkers' expression in EAC progression. In HNPCC, MMR protein mutation is associated with younger age of colorectal carcinoma onset (35), but we found no significant association between age or sex and MMR protein expression loss. Although advanced age and male sex are the risk factors for developing EAC, our data cannot support the use MSI immunohistochemistry in patients with advanced age and male sex as a prognostic marker for developing EAC.

P53 immunoreactivity has been reported in 53%-87% of patients with EAC, 55%-100% patients with HGD, 0%-71% patients with LGD, but not in intestinal metaplasia (36-41). Similar results were shown in our study, although with a high proportion of patients with p53 immunoreactivity in non-dysplasia group (86%). Significant but negative correlation was found only for MLH1/PMS2 expression and only in dysplasia group. This is to be confirmed in a larger cohort of patients but clearly shows that development of BE-associated adenocarcinoma is affected by many different pathways.

The most important limitation of this study is the low total number of patients and the low number patients with HGD, too low to be included in the statistical analysis. Also the study’s retrospective nature precludes us from concluding on the disease progression among patients with BE without dysplasia but with positive MMR mutation.

In conclusion, this is one of the first studies analyzing MSI in different stages of BE-EAC sequence. Although we presented interesting and potentially important data on the loss of MLH1/PMS2 expression in different stages of BE, due to small number of patients and retrospective analysis, we cannot conclude that MSI can be used as a biomarker to stratify the risk of EAC development. More prospective studies primarily following non-dysplastic BE patients with loss of MMR protein expression are needed.
